# Case Report: Long-term suppression of relapses by dimethyl fumarate in a relapsing pediatric patient with myelin oligodendrocyte glycoprotein antibody–associated disease manifesting as acute disseminated encephalomyelitis, unilateral optic neuritis, and seizure episodes

**DOI:** 10.3389/fimmu.2025.1551379

**Published:** 2025-02-25

**Authors:** Masayuki Tahara, Tomonari Awaya, Keisuke Saito, Hideyuki Sawada

**Affiliations:** ^1^ Department of Neurology, National Hospital Organization Utano National Hospital, Kyoto, Japan; ^2^ Department of Anatomy and Developmental Biology, Graduate School of Medicine, Kyoto University, Kyoto, Japan; ^3^ Department of Pediatrics, Graduate School of Medicine, Kyoto University, Kyoto, Japan

**Keywords:** myelin oligodendrocyte glycoprotein antibody-associated disease, dimethyl fumarate, acute disseminated encephalomyelitis, optic neuritis, seizure

## Abstract

**Background:**

Myelin oligodendrocyte glycoprotein (MOG) antibody-associated diseases (MOGAD), which has been recognized as a distinct entity in patients with neuromyelitis optica spectrum disorders, often presents with acute disseminated encephalomyelitis (ADEM) symptoms in pediatric patients. Appropriate treatment based on accurate diagnosis is challenging in relapsing pediatric patients with MOGAD.

**Case Presentation:**

An 11-year-old girl experienced relapses four times, exhibiting brainstem symptoms, an ADEM episode, seizures, and optic neuritis (ON). She was initially diagnosed with multiple sclerosis and received interferon beta-1a therapy with a mild effect on relapse suppression. She was then transferred from the pediatric department to the department of neurology of our hospital. Two months before her referral visit, she experienced left optic neuritis, and her annualized relapse rate reached 0.6. She desired to switch from the injectable treatment to oral dimethyl fumarate (DMF) administration. At that time, she was found to be seropositive for MOG antibody, but after that had no relapses for more than five years. Moreover, her seropositivity for serum MOG-antibody turned out to be seronegative.

**Conclusions:**

DMF showed long-term effects on suppressing relapses in a pediatric patient with MOGAD, revealing its potential as a treatment option for such patients.

## Introduction

In patients with neuromyelitis optica spectrum disorders (NMOSD) who were seronegative for aquaporin-4 antibody (AQP4-IgG), a new disease entity known as myelin oligodendrocyte glycoprotein (MOG) antibody-associated diseases (MOGAD), was proposed in 2023 (1). The clinical phenotype in MOGAD, including optic neuritis and myelitis, is consistent with that of AQP4-IgG-seropositive NMOSD; however, some differences in clinical characteristics, such as frequent pediatric onset, fewer relapses, and favorable recovery, have been noted.

Recently, the concept of MOGAD has been further broadened. Pediatric patients with MOGAD have an acute disseminated encephalomyelitis (ADEM) presentation, which often raises concerns about its differential diagnosis with multiple sclerosis (MS) (2), which is particularly problematic for determining the choice of treatment.

**Figure 1:**
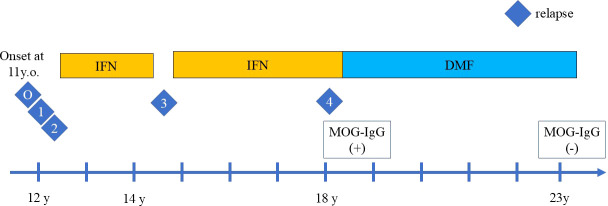
The clinical course of relapses during treatment with interferon beta-1a (IFN) injection and oral dimethyl fumarate (DMF). The relapses are indicated by the number. O indicates the disease onset, which manifested as ADEM with headache, vomiting, and hemiplegia followed by diplopia. One indicates the first relapse of ADEM episode with diplopia, right visual impairment, cerebellar symptoms, 2 indicates the second relapse with visual field impairment and left optic neuritis. IFN was then introduced, but the third relapse of focal seizure occurred during self-interruption of IFN, indicated by 3. Despite IFN treatment, the fourth relapse of left optic neuritis occurred, indicated by 4. After DMF induction, no relapses occurred.

Herein, we report the case of a relapsing pediatric patient with MOG antibody who responded well to dimethyl-fumarate (DMF), a drug indicated for MS, for more than five years. She experienced four relapses, which included brainstem symptoms, an ADEM episode, seizure, and unilateral optic neuritis. Prior to DMF treatment, she experienced two relapses before the induction of IFN beta-1a treatment, one relapse during self-interruption of IFN beta-1a, and the last relapse during IFN beta-1a treatment. Although she had a partial response to IFNbeta-1a, she had a better clinical course with DMF (**Figure 1**).

## Case report

An 11-year-old girl who was born healthy presented with headache, vomiting, and left hemiplegia. She had not been vaccinated before the onset of symptoms, but had a preceding infection with subtle rhinorrhea for one week. She was initially diagnosed with hemiplegic migraine. After half a month, she developed right eye abduction disorder and diplopia. Therefore, ADEM was diagnosed based on these symptoms of meningeal irritation followed by diplopia due to brainstem involvement. She was then referred to a nearby hospital, where brain magnetic resonance imaging (MRI) showed small lesions in the anterior horn of the right ventricle. A plain spinal MRI showed no apparent abnormalities. The examination of cerebrospinal fluid (CSF) showed pleocytosis, an elevation of myelin basic protein, but oligoclonal bands were not examined. The microbiological test of CSF was negative. The serological results showed no AQP4-IgG, but no measurement of MOG-IgG. She was treated with steroid pulse therapy, followed by oral steroid therapy for eight weeks.

**Figure 2:**
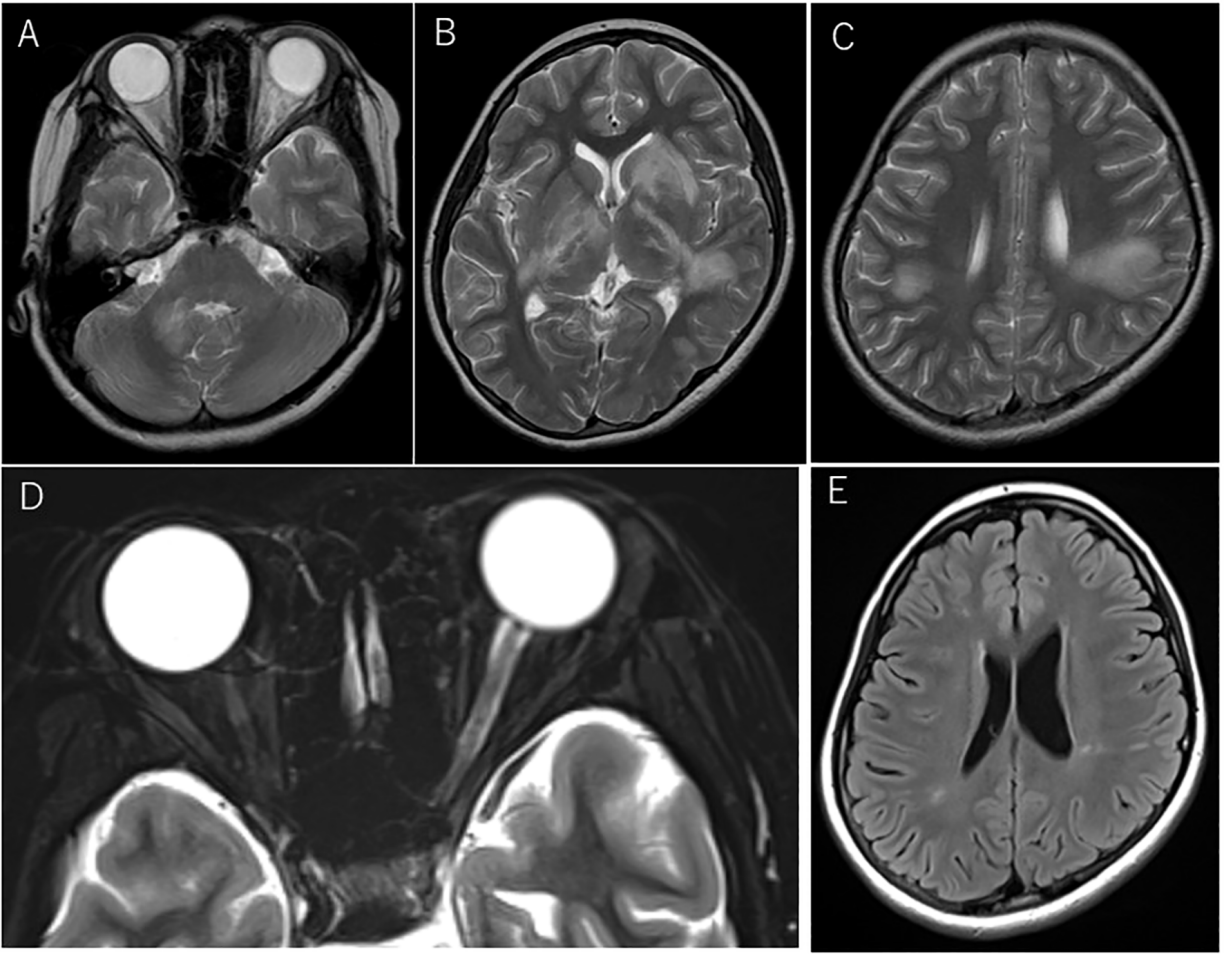
Magnetic resonance imaging (MRI) at acute disseminated encephalomyelitis (ADEM) and left optic neuritis relapses before dimethyl fumarate (DMF) administration. Axial T2-weighted MRI at the first relapse manifesting as ADEM **(A–C)**. Lesions involved the cerebellum**(A)** and a part of the basal ganglia**(B)**, including bilateral confluent lesions in the white matter **(C)**. At the fourth relapse, swelling of the left optic nerve and the intensity change of the optic nerve sheath were seen on axial T2-weighted images with fat suppression **(D)**. Shrinkage of multiple ADEM lesions was seen on FLAIR imaging at the referral visit to our hospital before the induction of oral DMF **(E)**.

Four months after the first episode, she developed diplopia with the restriction of her left eye abduction, followed by visual impairment in her right eye and cerebellar symptoms. Brain MRI revealed multiple white matter lesions (**Figures 2A–C**) and the examination of CSF was positive for oligoclonal band. Indicating multiple sclerosis (MS), she was treated with steroid pulse therapy for five days, which gradually improved her condition.

Two months later, she developed visual field impairment suggestive of inferior quadrantanopia and new white matter lesions involving right basal ganglia and right parietal lobe were found on brain MRI. The left relative afferent pupillary defect (RAPD) was positive. She then experienced decreased visual acuity on the left side, and the optic neuritis was confirmed with temporal pallor of the optic disc on the fundus examination and an enlarged left optic nerve on MRI, which prompted the administration of steroid pulse therapy. The interferon beta-1a (IFN) injection was initiated to suppress the relapse, but the treatment was discontinued after two years and two months at the patient’s request. At three months after discontinuation, she exhibited bilateral tonic clonic seizure that started from focal seizures in the left facial area. Long-term video-EEG monitoring was performed the day after the seizure, but no epileptogenic findings were seen. The test for NMDA receptor antibodies was not performed. She was then started on levetiracetam. The brain MRI showed no new lesions, but a follow-up brain MRI after one month indicated a new lesion, which prompted us to restart the IFN injection. The oral antiepileptic medication was discontinued within several months, with the patient remaining relapse-free for about three years and 7months. However, she suddenly developed visual impairment in her left eye decreased to 20/200 visual acuity, and her left RAPD was positive. Fundus examination revealed a slightly pallor of the optic disc and the left optic nerve on MRI showed the swelling except optic disc and intensity change of the optic nerve sheath in the orbit (**Figure 2D**) and faint gadolinium-enhancement in the optic canal, which was diagnosed as left optic neuritis. She was recovered rapidly in several days by steroid pulse therapy, followed by oral post-steroid therapy (tapering from a maximum dose of 1 mg/kg) for three months.

Two months later, she entered the university and was transferred from the pediatric department to our neurology department. At her first visit, no neurological abnormalities were observed, and a brain MRI showed mildly abnormal findings with shrinkage of the previous ADEM lesions involving the right cerebellar peduncle, bilateral basal ganglia, and multiple deep white matter lesions in the right temporal lobe and bilateral parietal lobes (**Figure 2E**), but no swelling of the bilateral optic nerves or laterality of signal intensity on MRI was observed. The annualized relapse rate until the referral visit was 0.6. Her neurological conditions and the MRI findings prompted us to switch her current treatment from IFN injection to oral dimethyl fumarate (DMF) administration, in line with the patient’s request. After starting DMF, blood tests revealed a high titer of anti-MOG antibody by cell-based assay, for which she was closely monitored by regular brain MRI at least every 6 months. The follow-up brain MRI showed no new lesions. Since then, however, no relapses have occurred for more than five years, with the disappearance of the anti-MOG antibody.

## Discussion

Biological drugs for treating AQP4-IgG-seropositive NMOSD have been extensively developed and widely used in recent years, with the primary targets being complement 5, IL-6, and B cells (3–7). On the other hand, the treatment of MOGAD, which was detected in patients with AQP4-IgG-seronegative NMOSD, has not yet been fully established.

DMF was indicated initially for psoriasis but was subsequently developed as a new drug for multiple sclerosis (8). Currently, DMF is commonly prescribed for patients with relapsing-remitting MS with mild disease activity and for pediatric patients with MS. However, there are only a few reports of patients with MOGAD treated with DMF. A case report suggested that DMF was ineffective in suppressing relapses in patients with MOGAD (9). In contrast, another case series showed that DMF was a potential trigger for the production of MOG antibodies in patients with MOGAD (10).

In a nationwide survey in Japan, only 44% of patients with MOGAD experienced relapses, and only some patients required treatment for relapse prevention (11, 12). However, sufficient therapy with rituximab or intravenous immunoglobulin (IVIG) upon disease onset (13) and treatment with prednisolone (12.5mg) for at least three months (14) could reduce the risk of subsequent relapses. In the current case, the patient received standard treatment, such as methylprednisolone steroid pulse therapy for the first attack, with no additional treatment.

Although MOG antibody titers at disease onset could not predict the disease course, seroconversion of MOG antibodies in relapsing patients has been associated with a decreased risk of relapse. Hence, conversion to seronegative during long-term treatment with DMF may support the patient’s relapse-free status in the future (15).

The clinical course of MOGAD mainly includes ADEM episodes, relapses of optic neuritis or myelitis, and infrequent brain symptoms, which may differ depending on whether the patient is aged over and under 18 years (16). Each clinical subtype may have varying pathophysiologies in patients with MOGAD. If the first symptom is an ADEM episode, a different diagnosis of MS may be complicated, requiring close monitoring. The patient in our case was indeed diagnosed with MS and was treated with IFN, which showed partial efficacy. However, she desired to discontinue the injections and instead receive oral treatment for MS.

Although our case showed a relapsing pattern and severe attacks, each attack displayed good recovery. In our case, optic neuritis occurred three times, once in the right eye and twice in the left eye. Although she did not show typical findings of MOGAD, such as bilateral optic neuritis or anterior involvement associated with optic disc edema, MRI showed not only swelling of the affected optic nerve but also involvement of the optic nerve sheath, which is known to be a unique finding in patients with MOGAD, distinct from multiple sclerosis and neuromyelitis optica spectrum disorder.

The clinical course of patients with MOGAD may not always be benign with good recovery (17). In relapsing patients with MOGAD who present with an aggressive and refractory course, another treatment option should be considered, and regular infusion of IVIG (18) might be one of the best choices, especially in steroid-dependent cases (19). IL-6 has also been identified as a potential target for MOGAD (20), and a clinical trial of IL-6 receptor blockade is currently ongoing.

In conclusion, our case suggests that DMF showed favorable long-term effects in suppressing relapses in a relapsing pediatric patient, highlighting its potential as a treatment option for patients with MOGAD.

## Data Availability

The original contributions presented in the study are included in the article/supplementary material. Further inquiries can be directed to the corresponding author/s.
